# Editorial: Glutamate-Related Biomarkers for Neuropsychiatric Disorders

**DOI:** 10.3389/fpsyt.2019.00904

**Published:** 2019-12-05

**Authors:** Chieh-Hsin Lin, Kenji Hashimoto, Hsien-Yuan Lane

**Affiliations:** ^1^Department of Psychiatry, Kaohsiung Chang Gung Memorial Hospital, Chang Gung University College of Medicine, Kaohsiung, Taiwan; ^2^Graduate Institute of Biomedical Sciences, China Medical University, Taichung, Taiwan; ^3^School of Medicine, Chang Gung University, Taoyuan, Taiwan; ^4^Division of Clinical Neuroscience, Chiba University Center for Forensic Mental Health, Chiba, Japan; ^5^Graduate Institute of Biomedical Sciences, China Medical University, Taichung, Taiwan; ^6^Department of Psychiatry & Brain Disease Research Center, China Medical University Hospital, Taichung, Taiwan; ^7^Department of Psychology, College of Medical and Health Sciences, Asia University, Taichung, Taiwan

**Keywords:** glutamate, NMDA, schizophrenia, depression, Alzheimer's disease, autoimmune encephalitis, autism, addiction

The glutamate receptor plays an important role in synaptic plasticity, learning, memory, and the pathogenesis and pathophysiology of numerous neuropsychiatric disorders such as schizophrenia, depression, addiction, autism, autoimmune encephalitis, and neurodegenerative diseases such as Alzheimer's disease.

For example, both ionotropic (especially NMDA and AMPA) and metabotropic (mGLU) receptors were reported to be involved in schizophrenia. In details, taking NMDA receptors (NMDARs) as an example, the receptors are widely distributed in most major organs (including heart, ovary, kidney, gastrointestinal system, lung, and etc.) and tissues (including ganglia cells, nerve fibers, blood vessels, enteroendocrine cells, liver, mast cells, inflammatory cells and etc.). The NMDAR has binding sites not only for glutamate or aspartate, but also a separate coagonist site for the endogenous ligands, D-serine, D-alanine, and glycine. Occupancy of the coagonist site can increase the frequency of opening of the channels activated by NMDAR agonists, facilitating excitatory neurotransmission in the brain. In fact, the binding of both glycine (or D-serine, D-alanine) and glutamate is required to open the NMDAR channel ionophore.

Some of the NMDARs isolated from peripheral tissues have been cloned and sequenced. These sequences correspond with NMDARs that have been cloned in the CNS. Further physiological and pharmacological experiments support the hypothesis that NMDARs in the periphery have similar properties to those in the CNS or expressed in host cells transfected with cloned subunits. Physiological studies with agonists and antagonists of the NR1 subunit of the NMDAR in the pig ileum have shown that these receptors are similar to those characterized in the CNS.

Direct measurement of physiological changes and NMDAR-related biomarkers in human brains is nearly impossible because of invasive testing procedures and ethical issues. Thus, developing accessible peripheral or brain image biomarkers become more important for mental illness. Peripheral gene expression may be a useful surrogate for gene expression in the CNS when the relevant gene is expressed in both. Lymphocytes or white cells have been suggested to be a neural probe because numerous studies showed similarities between receptor expression and mechanisms of transduction processes of cells in the nervous system (e.g. neurons and glia) and lymphocytes. Blood-derived RNA has also become a convenient alternative to traditional tissue biopsy-derived RNA. Therefore, peripheral gene expressions as well as other peripheral biomarkers might have potential to be surrogate CNS biomarkers for disorders, their outcomes, and treatment responses in the CNS.

Among the 12 articles of this research topic *Glutamate-Related Biomarkers for Neuropsychiatric Disorders*, the first paper *Elevated Glutamate and Glutamine Levels in the Cerebrospinal Fluid of Patients With Probable Alzheimer's Disease and Depression*, by Madeira et al., reports increased CSF levels of glutamate and glutamine in Alzheimer's disease and major depressive disorder compared to healthy controls and to patients with normal pressure hydrocephalus. Importantly, lower and Innotest amyloid tau index (IATI) scores were correlated with higher glutamate and glutamine levels in their study cohort. Further, in subjects without dementia (Mini-Mental State Examination [MMSE] above 23), lower MMSE scores were associated with higher glutamate and glutamine levels.

Accumulating evidence suggests that dysfunctions in in the glutamate-glutamine cycle also play a role in the pathophysiology of schizophrenia. Madeira et al. demonstrated altered blood levels of glutamate and glutamine in patients with schizophrenia in their another study, *Blood Levels of Glutamate and Glutamine in Recent Onset and Chronic Schizophrenia*. They indicate that circulating glutamine/glutamate ratios rise at the onset of illness but fall with progression of the disorder.

In a brief review, *Astrocytic Regulation of Glutamate Transmission in Schizophrenia*, Mei et al. updated findings on the crucial role of glutamate uptake and astrocyte-derived D-serine in the pathophysiology of schizophrenia. Neuroimaging and neurochemical biomarkers based on glutamate transmission and metabolism have also shown translational potential. Further elucidation of neuron-astrocyte interaction during the aforementioned processes will facilitate the development of novel diagnostic and therapeutic approaches for schizophrenia and related disorders.

Not only ionotropic but also metabotropic glutamate (mGlu) receptors are regarded as novel drug targets for the treatment of schizophrenia. Nicoletti et al. reviewed the development of selective ligands of individual mGlu receptors as new therapy for schizophrenia in the article *Targeting mGlu Receptors for Optimization of Antipsychotic Activity and Disease-Modifying Effect in Schizophrenia*. Subtype-selective mGlu receptor ligands thereby offer the potential of a precision medicine based pharmacological approach for various domains of schizophrenia. Moreover, some mGlu receptor ligands may exert a positive influence on neuroinflammation by modulating microglial function or other mechanisms.

While glutamate is the agonist of the NMDAR, D-serine appears to be the most potent coagonist. The review article (*D-Serine: Potential Therapeutic Agent and/or Biomarker in Schizophrenia and Depression?*) by Mackay et al. indicates that neurons are more relevant to D-serine action than originally thought. Interestingly, D-serine appears to have antipsychotic and antidepressive properties. D-serine may be also a biomarker for antidepressant response to ketamine. Preclinical and clinical studies reveal that D-serine may be a potential cognitive enhancer, deserving further study.

Besides D-serine and D-alanine, another endogenous D-amino acid in the mammalian brain, D-aspartate, can influence NMDAR-mediated transmission. Errico et al. in their review, *The Emerging Role of Altered D-Aspartate Metabolism in Schizophrenia: New Insights From Preclinical Models and Human Studies*, indicated that increased levels of the NMDAR agonist, D-aspartate, impact on a series of functional and structural phenotypes relevant to schizophrenia. Moreover, these findings suggest a possible role of dysregulated embryonic D-aspartate metabolism in schizophrenia pathogenesis, and consequently propose the potential of D-aspartate as a novel therapy or prevention for this neurodevelopmental brain disorder.

D-amino acid oxidase activator (DAOA, also known as G72) protein regulates D-serine neurotransmission. Previous study found that the peripheral G72 protein expression is distinctively higher in patients with schizophrenia than in healthy individuals. Lin and Lin in their study *Combination of G72 Genetic Variation and G72 Protein Level to Detect Schizophrenia: Machine Learning Approaches*, demonstrated that G72 protein alone, even without adding G72 SNPs, can discriminate schizophrenia patients from healthy subjects, with adequate power. They also suggest a combination of logistic regression and naive Bayes models to establish an optimal model to predict schizophrenia.

Dysfunctional glutamatergic neurotransmission is implicated in the etiology of not only schizophrenia but also major depressive disorder. In the review article *Glutamatergic Dysfunction and Glutamatergic Compounds for Major Psychiatric Disorders: Evidence From Clinical Neuroimaging Studies*, Li et al. indicated that ketamine, an NMDAR antagonist, has displayed its rapid antidepressant efficacy for treatment-resistant depression. Many compounds which modulate the glutamatergic transmission have also shown potential in the treatment of major psychiatric disorders, via approaches such as magnetic resonance spectroscopy, positron emission tomography/single-photon emission computed tomography, and paired-pulse transcranial magnetic stimulation.

On the other hand, ketamine is also a common drug of abuse, while disrupted oxytocin system is involved in the development of addiction. In the original study *Decreased Blood Levels of Oxytocin in Ketamine-Dependent Patients During Early Abstinence*, Huang et al. found that the oxytocin level was significantly lower in ketamine-dependent patients and the level did not normalize after early abstinence. Lower oxytocin might be also related with anxious phenotype of ketamine dependence. These results suggest the potential of therapeutic use of oxytocin for treating ketamine dependence.

Previous studies have demonstrated that plasma asymmetric dimethylarginine (ADMA) was increased in patients with schizophrenia and correlated with cognitive dysfunction. In the study *Treatment Responses of Cognitive Function and Plasma Asymmetric Dimethylarginine to Atypical Antipsychotic in Patients With Schizophrenia*, Yu et al. found that atypical antipsychotic treatment in acutely exacerbated schizophrenia patients improved psychiatric symptoms and cognitive function (especially working memory and attention), in parallel with decreased plasma ADMA levels, implying that plasma ADMA may be a potential indicator of cognitive recovery in schizophrenia.

In the review, *Autism Associated With Anti-NMDAR Encephalitis: Glutamate-Related Therapy*, Tzang et al. addressed the role of autoimmune dysfunction in autism. The anti-NMDAR autoantibody can lead to dysfunctional glutamate neurotransmission in the brain that manifests as various psychiatric symptoms such as psychosis and personality changes. Glutamate-related therapy primarily normalizes glutamate neurotransmission and can be a new adjuvant intervention alongside antipsychotics for treating autoimmune autism.

Acupuncture has been regarded as a complementary therapy for neuropsychiatry disorders. In the article, *The Effects of Acupuncture on Glutamatergic Neurotransmission in Depression, Anxiety, Schizophrenia, and Alzheimer's Disease: A Review of the Literature Review*, Tu et al. reported the current evidence for the treatment efficacy of acupuncture in depression, anxiety, schizophrenia, and Alzheimer's disease. Further, they discuss the ways in which acupuncture treatment can potentially modulate glutamate receptors and excitatory amino acid transporters.

Overall, to promote the development of glutamate-related biomarkers for neuropsychiatric disorders, this research topic gathers a comprehensive body of review and original articles to update the current state of this increasingly important theme. Further basic, preclinical and clinical studies are needed to elucidate the possible mechanisms of glutamate-related dysfunction in neuropsychiatric disorders and to establish clinical applications for diagnoses and treatments of these disorders.

## Author Contributions

C-HL and H-YL wrote the first draft of the manuscript, and KH provided opinions on it. All authors read and approved the submitted version.

## Funding

This work was partly supported by Ministry of Science and Technology, Taiwan (MOST 107-2632-B-039-001; 108-2314-B-039-002; 108-2622-B-039-001-CC2; 108-2628-B-182A-002), National Health Research Institutes (NHRI-EX108-10731NI; NHRI-EX108-10816NC), China Medical University Hospital, Taiwan (DMR-105-076), Chang Gung Memorial Hospital, Taiwan (CMRPG8G1391), and Taiwan Ministry of Health and Welfare Clinical Trial and Research Center of Excellence (MOHW108-TDU-B-212-133004).

## Conflict of Interest

The authors declare that the research was conducted in the absence of any commercial or financial relationships that could be construed as a potential conflict of interest.

